# Preventing Photomorbidity in Long-Term Multi-color Fluorescence Imaging of *Saccharomyces cerevisiae* and *S. pombe*

**DOI:** 10.1534/g3.120.401465

**Published:** 2020-10-06

**Authors:** Gregor W. Schmidt, Andreas P. Cuny, Fabian Rudolf

**Affiliations:** * ETH Zurich, Department of Biosystems Science and Engineering, Mattenstrasse 26, 4058 Basel, Switzerland and; * SIB Swiss Institute of Bioinformatics, 4058 Basel, Switzerland

**Keywords:** photomorbidity, phototoxicity, fluorescence, microscopy, live-cell, imaging, long-term, time-lapse, quantitative, yeast

## Abstract

Time-lapse imaging of live cells using multiple fluorescent reporters is an essential tool to study molecular processes in single cells. However, exposure to even moderate doses of visible excitation light can disturb cellular physiology and alter the quantitative behavior of the cells under study. Here, we set out to develop guidelines to avoid the confounding effects of excitation light in multi-color long-term imaging. We use widefield fluorescence microscopy to measure the effect of the administered excitation light on growth rate (here called photomorbidity) in yeast. We find that photomorbidity is determined by the cumulative light dose at each wavelength, but independent of the way excitation light is applied. Importantly, photomorbidity possesses a threshold light dose below which no effect is detectable (NOEL). We found, that the suitability of fluorescent proteins for live-cell imaging at the respective excitation light NOEL is equally determined by the cellular autofluorescence and the fluorescent protein brightness. Last, we show that photomorbidity of multiple wavelengths is additive and imaging conditions absent of photomorbidity can be predicted. Our findings enable researchers to find imaging conditions with minimal impact on physiology and can provide framework for how to approach photomorbidity in other organisms.

Obtaining a detectable signal in fluorescent live-cell imaging requires a bright probe and sufficient excitation light. However, excitation light can also influence cell physiology as it is absorbed and excites a variety of cellular molecules (Cadet *et al.* 2012) in addition to the fluorescent species of interest. For example, short wavelength light (<330 nm) is absorbed by DNA creating mutagenic cyclobutane pyrimidine dimers (Sancar 2016). Microscopists typically prevent such mutagenic damage by using UV blocking filters when illuminating with arc lamps, or by utilizing light sources which do not emit UV light (Frigault *et al.* 2009). In contrast, UV-A and visible light (>330 nm) can cause cellular damage through indirect mechanisms (Kielbassa *et al.* 1997; Godley *et al.* 2005). Most prominently, the cell physiology is altered by creating reactive oxygen species (ROS) originating from the excitation of endogenous (*e.g.*, metabolites) or exogenous (*e.g.*, media components) light absorbing molecules, collectively termed photosensitizers (Cadet *et al.* 2012). ROS subsequently form adducts with nucleotides, proteins or other metabolites altering or interfering with their function (Godley *et al.* 2005; Cadet *et al.* 2012). However, as ROS are generated as a bi-product of normal cell physiology, cells are competent in clearing a limited amount of light-induced ROS damage, and therefore a threshold for visible light dose exists below which no damage is detectable (Tinevez *et al.* 2012).

Initial attempts measured the mitotic arrest in a tobacco cell line (Dixit and Cyr 2003) and morphological changes and cell death in mammalian cells (Magidson and Khodjakov 2013). These are terminal phenotypes and as such not suited to detect subtle changes in cell physiology. Subtle light-induced effects have been detected in different cell types as changes in cell migration, chromosome movements or mitochondrial membrane potential as well as changes in the proliferation cells or development of embryos (Icha *et al.* 2017). In *Saccharomyces cerevisiae*, photodestructive effects were measured using the frequency changes of the nuclear localization of the general stress activated transcription factor Msn2 as a quantitative trait (Jacquet *et al.* 2003; Logg *et al.* 2009). As expected, a threshold light dose was found below which Msn2 localization frequency remained unchanged (Logg *et al.* 2009). However, this assay is very specific to the *Saccharomyces sensu stricto* and it is unclear if the localization frequency of Msn2 shows an equal sensitivity across the light spectrum. An additional complication of any fluorescent assay is that the sensitivity of the fluorescent readout is directly related to the administered light dose and can be distorted by photobleaching.

How can the threshold light dose be determined? A generic measure of cellular well being of dividing cells is the growth rate (GR). It has been used as a sensitive, quantitative read-out in diverse applications such as chemogenetic profiling (Lee *et al.* 2014) and systematic mapping of genetic interaction networks (Dixon *et al.* 2009). Moreover, changes in growth, measured as the timing of cell division, were shown to allow for quantifying subtle, non-toxic effects induced by fluorescence excitation light in *C. elegans* (Tinevez *et al.* 2012), mammalian cell culture (Laissue *et al.* 2017) and also *S. cerevisiae* (Carlton *et al.* 2010). We therefore reasoned that the growth rate of dividing cells can be employed as a measure for the absence of photo-destructive effects.

Here, we set out to develop guidelines to avoid the confounding effects of visible light in multi-color long-term fluorescence imaging using the model organisms *S. cerevisiae* and *S. pombe*. We aimed to measure ”photomorbidity”, which is defined as the effects of light on cellular well being as measured by decreased growth rate compared to an unstressed control. This is consistent with the definition of ”morbidity” by IUPAC: ”departure, subjective or objective, from a state of physiological or psychological well-being” (IUPAC 2016) and distinct from the more severe term ”phototoxicity”, which is used to describe cell death. We showed that photomorbidity can be described using a classical dose-effect relationship with a characteristic effective dose (ED_50_) and a no-observed effect level (NOEL) below which confounding effects are not detectable. We found that both measures depend on the cumulative light dose, and within practical boundaries are independent of the imaging interval, light intensity and bandwidth. To compare the suitability of different imaging channels and fluorescent proteins for live-cell imaging, we determined the signal-to-noise ratio (SNR) obtained with fluorescent fusion proteins at the NOEL of their specific excitation wavelength. Our results demonstrate how photomorbidity and cellular autofluorescence, in addition to fluorescent protein brightness, can influence the performance of fluorescent proteins in live-cell imaging. We found that photomorbidity of combined wavelengths in multi-color imaging was additive and could be avoided by limiting light doses to the NOEL. In addition, our findings highlight how particular combinations of fluorescent reporters enable artifact free multi-color time-lapse imaging.

## Materials and Methods

### Yeast culturing

*S. cerevisiae* cells were precultured in either synthetic minimal medium (Smin containing 1.7 g/l yeast nitrogen base without amino acids or ammonium sulfate (BD Biosciences, Germany), 5 g/l ammonium sulfate) or YP medium (10 g/l yeast extract, 20 g/l peptone, both BD Biosciences, Germany) while *S. pombe* cells were grown in Edinburgh minimal medium (6.77 g/l EMM-nitrogen-glucose minimal media (Sunrise Science Products, San Diego, USA), 5 g/l ammonium sulfate) at 30 or 32° in an orbital shaker (Sherman 2002; Moreno *et al.* 1991). All media were supplemented with 2% (w/v) of the appropriate carbon source. All chemicals were purchased from Sigma-Aldrich Co. (Germany), unless stated otherwise. Cells were innoculated from freshly streaked plates in 5 mL medium one (glucose) to two days (acetate and glycerol) before the experiment. The evening before the start of the experiment, cells were diluted 1:500 (glucose) or 1:50 (acetate and glycerol) in fresh media. For loading of the microfluidic device, the cell concentration was measured using a Z2 Coulter Counter (Beckman Coulter, Nyon, Switzerland). Typical cell concentrations ranged from 0.5*10^6^ to 5*10^6^ cells/ml. 1 mL of culture was transferred to a 1.5 mL tube and spun at 1000 g for 2 min and the appropriate amount of supernatant was removed to yield a final cell concentration of 1*10^7^ cells/ml. The cells were resuspended in the remaining media using a Vortex Genie 2 (Scientific industries, New York, USA) for 10 sec at speed setting four.

### Plasmid construction

All plasmids were constructed using classical DNA manipulation techniques. Restriction enzymes and T4 DNA ligase were purchased from New England BioLabs. DNA sequences were amplified using Phusion High-Fidelity DNA Polymerase (Thermo Scientific). Yeast codon optimized fluorescent protein sequences were derived from pKT139 (Citrine A206K) (Sheff and Thorn 2004), used in a previous work (sfGFP) (Roberts *et al.* 2016) or synthesized from GeneArt (mTFP1, mAmetrine, mCardinal, mNeptune2, mNeongreen, mTurquoise2, mKO*κ*, tSapphire, mRuby2, mKate2, tdKO*κ*). All fluorescent proteins were cloned into pKT102 using PacI/AscI flanking restriction enzymes (Sheff and Thorn 2004). eGFP in the pFA6 backbone was obtained from Addgene #44900 (Lee *et al.* 2013). All plasmid sequences were checked by Sanger sequencing (Microsynth AG, Switzerland). The plasmids are listed in Table S8.

### Yeast strain construction

All *S. cerevisiae* photomorbidity experiments were carried out using a prototrophic FY4 strain (FRY2032). FRY2032 was obtained by mating of BY4700 and BY4707 (Brachmann *et al.* 1998) and subsequent sporulation (parent strain FRY2023) and selection on minimal media (Smin + glucose). All *S. pombe* experiments were carried out using a prototrophic 972 h- strain (FRSP902) (Leupold 1950). For fluorescent protein characterization, all *S. cerevisiae* strains were derived by endogenous tagging of the prototrophic strain FRY1455 (Gnügge *et al.* 2016). Tagging cassettes were amplified from the modified pFA6 cassettes (Table S8) by polymerase chain reaction (PCR), using Phusion High-Fidelity DNA Polymerase (Thermo Scientific, USA). Yeast transformations were performed using the Lithium-Acetate method (Daniel Gietz and Woods 2002) and successful integration was checked by fluorescence microscopy and/or colony PCR. Table S9 lists all yeast strains used in this work. Table S7 lists the primers used for tagging/knock-out/colony PCR of Vph1, Cdc12 and Whi5.

### Yeast colony PCR

We checked integration at the targeted site by a colony PCR assay. Yeast colonies were boiled in 3 *μ*l 20 mM NaOH for 10 min and DNA was amplified in a 25 *μ*l volume with 1 M betaine, 1x ThermoPol buffer (New England BioLabs), 0.2 mM each dNTP, 2 *μ*M each primer (Table S7) and 1.25 U Taq DNA Polymerase (New England BioLabs), using the following PCR program: 5 min initial denaturation at 94°; 30 cycles of 30 s at 94°, 30s at 58°, 1 min/kbp at 72°; and 10 min final elongation at 72°.

### Microfluidic chip fabrication

The microfluidic chip (adapted from (Frey *et al.* 2015)) was designed in AutoCAD (Autodesk, München, Germany). The chip consists of one layer of polydimethylsiloxane (PDMS, Sylgard 184, Dow Corning Corp., USA), attached to a 150-*μ*m-thick cover glass (24 mm × 60 mm) using vacuum. The PDMS layer was cast from 4-inch silicon wafers patterned using standard photolithography and dry etching processes. In short, the first layer was fabricated by patterning a dry etching mask using ma-P 1240 photoresist (micro resist technology GmbH, Berlin-Koepenick, Germany) according to the manufacturers instructions. Dry etching was performed on an Ionfab 300 (Oxford instruments, Abingdon, United Kingdom). The first layer defines the gap between the glass and the clamping pad and its height was modified for the specific yeast type: 3.8 *μ*m for *S.cerevisiae* and 4.1 *μ*m for *S.pombe*. Three layers of SU-8 were patterned on top of the dry etched silicon substrate to complete the microfluidic design. We followed the datasheet of the manufacturer (Microchem Corp.,Westborough, USA) to structure each of the SU-8 layers. In brief, SU-8 was spin-coated at the desired thickness and soft-baked on a hotplate. The SU-8 was then exposed through a transparency mask (Selba S.A., Versoix, Switzerland) using a UV mask aligner (MA/BA8-Gen3 mask aligner, SUSS MicroTec AG, Garching, Germany) and baked on a hotplate for cross-linking. Unexposed SU-8 was removed using mr-Dev600 (micro resist technology GmbH, Berlin-Koepenick, Germany). The molds were coated with trichloro (^1^H,^1^H,^2^H,^2^H-perfluoro-octyl)silane (Sigma-Aldrich, Switzerland) in a vapor silanization process, for at least one hour. 25g of freshly mixed and degassed polydimethylsiloxane (PDMS Sylgard 184, 10:1 (w/w) monomer:curing agent) was poured onto the mold. After curing in a convection oven at 80° for at least 2 h, the PDMS was released from the silicon wafer. Single chips were cut from the PDMS sheet and access holes were punched at inlet and outlet sites.

### Setup of microfluidic experiment

The PDMS device and glass slide were rinsed with acetone, isopropanol, deionized water and dried using a nitrogen gun. The PDMS device was placed with its structured side facing up in a sterile cell culture hood for chip loading. 0.4 *μ*l of the cell solution was dispensed onto each culturing area of the microfluidic device by using a conventional pipette. The cover glass was placed on top of the device such that it sealed the microfluidic channels. The cover glass and device were held together by adhesion. The device was then transferred to the microscope. First, the vacuum channel was attached to the in-house vacuum supply to facilitate a rigorous adhesion of the PDMS chip to the glass slide using microfluidic tubing (Tygon LMT-55, inner diameter: 0.51 mm, wall thickness: 0.85 mm). Next, the inlet and outlet tubing (Tygon LMT-55, inner diameter: 0.51 mm, wall thickness: 0.85 mm) was attached and the flow through the device (10 *μ*l/min) was started. The flow was established by syringe pumps (Nemesys, CETONI GmbH, Korbussen, Germany) using medical glass syringes (PTFE TLL Luer Lock 25 mL syringe, Innovative Labor Systeme GmbH, Stuetzerbach, Germany). Bubbles introduced during loading were removed in less than one hour through diffusion through the PDMS into the vacuum channel which surrounded the fluid channels. The microfluidic device was placed in a custom-made aluminum chip holder, and fixed using nail polish (Maybelline, L’Oreal Suisse S.A, Vernier, Switzerland).

### Measurement of microscope excitation light spectra and intensity

To measure the light spectra of the fluorescence and transmitted illumination light source, an Ocean Optics USB2000+ spectrometer (Ocean Optics, Dunedin, USA) was used. The optical fiber of the spectroscope was inserted into a 5mm thick block of PDMS (Sylgard 184, Dow Corning, Midland, USA) plasma bonded to a 170 *μ*m cover slide and brought into focus of the objective. The light from the fluorescence excitation light source (Spectra X Light Engine, Lumencor, Beaverton, USA) was transmitted through a 40x Plan Fluor Oil DIC N2 NA 1.3 objective (MRH01401, Nikon). To avoid stray light influencing the measurement, the aperture diaphragm was used to restrict illumination to the opening of the optical fiber only. To reduce the light intensity, two neutral density filters (Cyto 2.0 ND 200-0065) were mounted into the same filter cube as the dichroic. The respective excitation filter - dichroic combination (see Table S3 & S6) was inserted in the light path and the light intensity setting of the respective LED was set to 100%. For the transmitted illumination light source the glass slide with the PDMS and optical fiber was turned upside down. The condenser of the microscope was used to focus the transmitted lightsource onto the optical fiber and the field diaphragm was closed to illuminate only the fiber opening. Raw spectra were recorded using Spectra Suite (Ocean Optics, Dunedin, USA). The integration time of the sensor was adjusted so that the peak intensity was between 4-6*10^4^ AU (5-200 ms). Dark spectra with the same integration time and the light source turned off were recorded for background correction. Data were exported as comma delimited file and Matlab (MathWorks, Natick, USA) was used for background correction and calculation of the center wavelength.

The light intensity of the epifluorescence excitation light was measured similarly to what was described earlier (Grünwald *et al.* 2008). In short, the field diaphragm of the epifluorescence light path was adjusted such that the diaphragm was visible in the field of view of the camera, using the 40x Plan Fluor Oil DIC N2 NA 1.3 objective (MRH01401, Nikon Instruments AG, Egg, Switzerland) and a fluorescent Argo-M Standard microscopy slide (Argolight, Talence, France). An image of the field diaphragm was taken and the number of illuminated pixels was determined in ImageJ. The illuminated area in the sample plane was calculated from the number of illuminated pixels, the size of the pixels of the camera (6.5 *μ*m × 6.5 *μ*m) and the magnification of the objective, Next, the objective was removed and an adjustable iris (SM1D12C, Thorlabs, Newton, USA) was mounted in its place. The diameter of the iris was adjusted to restrict the light beam to the diameter of the objective back aperture, to measure only the light that is actually entering the objective. An S170C microscope slide power sensor (Thorlabs, Newton, USA) was placed in the microscope stage holder, and the power was measured using a PM100USB power meter (Thorlabs, Newton, USA). The respective excitation filter - dichroic combination (see Table S3 & S6) was inserted in the light path and the previously determined central wavelength for each filter set was entered in the PM100USB software, to acquire wavelength corrected power measurements. The light power was measured for light intensity settings of the respective LED ranging from 10% to 100% in steps of 10%. The data were exported as a comma delimited file. Matlab (MathWorks, Natick, USA) was used for calculating the light intensities at the sample plane.

### Measurement of the exact exposure time of the microscope setup

To measure the actual time a sample is exposed to the excitation light, we used a TSL2561 (AMS-TAOS USA Inc.) light to digital converter on a SparkFun Luminosity Sensor Breakout board (SparkFun Electronics, USA). The sensor was connected to an Arduino UNO SMD R3 (Arduino LLC) via the SDA and SCL channels and powered by the Arduino UNO board internal 3.3V power supply. A self-made holder was used to mount a glass fiber (diameter: 50 *μ*m, D+X Produkte GmbH, Turbenthal, Switzerland) directly above the photo-active area of the TSL2561 using a duplex glass fiber adapter. The other end of the glass fiber was glued into a Luer Lock needle tip. The needle tip with the glass fiber was inserted into a PDMS block (Sylgard 184, Dow Corning, Midland, USA), which was plasma bonded to a 170 *μ*m cover glass. The PDMS/glass sandwich was placed on the microscope and the glass fiber brought into focus of the 40x Plan Fluor Oil DIC N2 NA 1.3 objective (MRH01401, Nikon). The integration time of the TSL2561 light sensor was set to the shortest possible period (13.7 ms). Exposure times of 50, 100, 200, 500, 1000 and 2000 ms were applied five times each using the microscope control software YouScope and the luminosity was recorded continuously. Matlab (MathWorks, Natick, USA) was used to extract the actual exposure times from the luminosity measurements and plot the measured values against the set exposure times. A linear function was fitted to the data (Figure S4). On average, the additional exposure time due to hardware delays was 187 ms and was constant over all set exposure times. The additional exposure time was taken into account when calculating the light doses applied during photomorbidity measurements.

### Microscopy

We chose a microscope configuration which allowed for maximum detectability of the fluorescent signal. Unless stated otherwise, we used a 40x Plan Fluor Oil DIC N2 objective with an NA of 1.3 (MRH01401, Nikon Instruments AG, Egg, Switzerland), as this allowed the maximum SNR (SNR ∼ NA^4^* Magnification^−2^, (Rines *et al.* 2011; Frigault *et al.* 2009)) to be obtained while maintaining subcellular resolution and imaging of the whole cellular volume in a single focal plane.

Experiments were performed on a Nikon Ti Eclipse (Nikon Instruments AG, Egg, Switzerland) inverted fluorescence microscope controlled using YouScope (Lang *et al.* 2012). The microscope was placed in an environmental enclosure (Life Imaging Services, Switzerland) to maintain the desired temperature. To keep the cells in focus over the time course of the experiment the Perfect Focus System was enabled. Images were recorded using an ORCA Flash 4.0 V1 camera (Hamamatsu Photonic, Solothurn, Switzerland) operated in water cooling mode (20°). Unless stated otherwise, the microscope was equipped with a Spectra X Light Engine fluorescence excitation light source (Lumencor, Beaverton, USA) and a pE-100 brightfield light source (CoolLED Ltd., UK). For multi-color NOEL imaging, hardware triggering between the light sources and the camera was implemented using an Arduino UNO (Somerville, MA, USA). All measurements were run with a diffuser and a green interference filter placed in the brightfield light path.

The optical filters and light intensities used for the measurement of photomorbidity can be found in Table S3. The optical filters and light intensities used for the measurement of the SNR of the fluorescent protein fusions can be found in Table S6. All optical filters were purchased from AHF Analysetechnik AG (Tuebingen, Germany).

### Image analysis

For cell segmentation out-of-focus brightfield images were acquired (± 5 AU, Nikon Perfect Focus System). As an input for cell segmentation brightfield images acquired above the focal plane were divided by brightfield images acquired below the focal plane. The division of images leads to elimination of uneven illumination and enhances the membrane diffraction pattern of the individual cells yielding a better segmentation. Image division was carried out using the ”Image Calculator” function of ImageJ. Cell segmentation was performed using CellX (Mayer *et al.* 2013). Fluorescent properties were extracted using the in-built functions of CellX.

### Measurement of photomorbidity

The microfluidic chip was loaded as described earlier. For morbidity measurements the cells were atoned on the chip for 3-4 hr before time-lapse imaging was started. Imaging was performed in five minute intervals, unless stated otherwise. For measuring one morbidity curve, the light dose was altered by adjusting the exposure time and at least six different light doses were tested in each experiment. A control measurement, where cells were not exposed to any epifluorescence excitation light was included in each measurement. For each light dose and the control, five independent positions in the chip were imaged. Hence, each photomorbidity curve consists of at least 35 data points (measured culturing pads). Some photomorbidity experiments were repeated with different exposure times to capture the whole dose-effect relationship. The optical filters and light intensities used for the measurement of photomorbidity can be found in Table S3. During calculation of the applied light doses the hardware delays during exposure were taken into account. Photomorbidity experiments were performed at 30° in Smin + glucose media for *S. cerevisiae* and at 32° and in EMM + glucose media for *S. pombe*, unless stated otherwise. Time-lapse imaging was carried out for 495 min (100 iterations). In the resulting images the regions occupied by colonies where cells touch the image border at any time of the experiment were excluded using in ImageJ (National Institute of Mental Health, Maryland, USA) before segmentation. The number of cells was subsequently extracted by segmentation using CellX (see Image analysis for details). The growth rate was calculated as follows:

$$GR=\frac{log_{2}\frac{Cells_{\left(495min\right)}}{Cells_{\left(0min\right)}}}{495min}$$ (1)

The calculation of the growth rate was based solely on the first (Cells(0min)) and last (Cells(495min)) image of the observation period, and gives the average growth rate over the whole observation time. This facilitates the growth rate measurement in cases where the growth rate changes over time. Also, the average growth rate is in good agreement with growth rates calculated from cell numbers determined every five minutes, where the growth rate was determined by fitting an exponential growth model. For *S. cerevisiae* the doubling time (doubling time = 60 min/growth rate) is 117 ± 6 min if all datapoints are fitted with an exponential model and 123 ± 7 min if the average doubling time is calculated from the first and last image only. Likewise, for *S. pombe* fitting of all datapoints yielded doubling times of 130 ± 6 compared to 128 ± 10 min calculated from the first and last image.

The growth rates were plotted against the light dose and the dose-effect model was fitted using the Matlab R2015b ”fit” function (Mathworks, Natick, USA).

$$GR_{\left(D\right)}=\frac{GR_{\left(0\right)}}{1+{\frac{LD}{ED_{50}}}^{\tau }}$$ (2)

The residuals were minimized using a robust estimate relying on the absolute distance. The 95% confidence interval of the ED_50_ and *τ* were obtained from the fit parameters using the ”confint” function. The no-observed effect level (NOEL) was extracted from the fit as the light dose (LD) at which the growth rate is reduced to 98% of the control growth rate (GR(0)).

### Measurement and calculation of fluorophore brightness and signal-to-noise ratio

Where possible, the SNR is determined from image areas which contain a homogeneous distribution of signal ($\mu_{sig}$) and background ($\mu_{bg}$). However, in biological samples signal and background are non-homogeneously distributed. We therefore determined the signal ($\mu_{sig}$) by measuring endogenously tagged cells and the noise ($\mu_{bg}$) by measuring a parent strain carrying no fluorescent protein tag. To analyze a large number of cells, *S. cerevisiae* cells were loaded in the microfluidic chip and grown at 30° in Smin + glucose media for at least 12 h. The intensity of the fluorescence excitation light was adjusted to 2.91 W cm^−2^ h^−1^ for all imaging channels. The fluorescent proteins were imaged as endogenous fusions to either Vph1, Cdc12 or Whi5. Images at different exposure times were taken to measure the light dose dependent average signal value $\mu_{sig}$ ($\mu_{vph1}$, $\mu_{cdc12}$ and $\mu_{whi5}$). A parent strain carrying no fluorescent protein tag was imaged under the same conditions to measure $\mu_{bg}$. To exclude effects of photobleaching, each position on the chip was imaged only once.

The obtained images were segmented using CellX (see Image analysis for details) and the segmentation mask was applied to the fluorescence images. $\mu_{sig}$ and $\mu_{bg}$ were extracted as the mean intensity value of the pixels which were identified as belonging to cells.

The measured SNR (mSNR) was calculated as:

$$mSNR_{\left(LD\right)}=\frac{\mu_{sig\left(LD\right)}-\mu_{bg\left(LD\right)}}{\sqrt{\mu_{sig\left(LD\right)}}}$$ (3)

For the calculation of the mSNR, the measurements at discrete light doses (LD) were used.

To be able to predict the SNR (pSNR) for any light dose, $\mu_{sig}$ and $\mu_{bg}$) and their respective standard deviations were plotted against the applied light dose. Linear functions (”ax + b”) were fitted using the ”weightedfit” function of Matlab R2015b (Mathworks, Natick, USA). From the parameters of the fits, $\mu_{sig}$ and $\mu_{bg}$ could be predicted for any light dose. For Vph1 $\mu_{sig}$ ($\mu_{vph1}$) was directly accessible from the images acquired from the respective Vph1-tagged strains. For Cdc12 and Whi5, $\mu_{sig}$ ($\mu_{cdc12}$ and $\mu_{whi5}$) was estimated based on the signal levels of the Citrine tagged Vph1, Cdc12 and Whi5 reference strains as follows:

$$\mu_{cdc12_{FP}}=\left(\mu_{vph1_{FP}}-\mu_{bg_{FP}}\right)\text{*}\left(\frac{\mu_{cdc12_{Citrine}}-\mu_{bg_{Citrine}}}{\mu_{vph1_{Citrine}}-\mu_{bg_{Citrine}}}\right)+\mu_{bg_{FP}}$$ (4)

where,

$$\left(\frac{\mu_{cdc12_{Citrine}}-\mu_{bg_{Citrine}}}{\mu_{vph1_{Citrine}}-\mu_{bg_{Citrine}}}\right)=3.33$$ (5)

and,

$$\mu_{whi5_{FP}}=\left(\mu_{vph1_{FP}}-\mu_{bg_{FP}}\right)\text{*}\left(\frac{\mu_{whi5_{Citrine}}-\mu_{bg_{Citrine}}}{\mu_{vph1_{Citrine}}-\mu_{bg_{Citrine}}}\right)+\mu_{bg_{FP}}$$ (6)

where,

$$\left(\frac{\mu_{whi5_{Citrine}}-\mu_{bg_{Citrine}}}{\mu_{vph1_{Citrine}}-\mu_{bg_{Citrine}}}\right)=11.1$$ (7)

Using the extrapolated values for $\mu_{sig}$, the predicted SNR (pSNR) for each fluorescent protein fusion at any light dose could be calculated according to equation 3.

The fluorophore brightness relative to eGFP was calculated based on the linear fit parameters, when $\mu_{sig}$ and $\mu_{bg}$ were plotted against the light dose, as described earlier. The brightness calculation was based on the signal from the Vph1-tagged strains ($\mu_{vph1}$):

$$Brightness_{FP}=\frac{\mu_{vph1_{FP}}-\mu_{bg_{FP}}}{\mu_{vph1_{GFP}}-\mu_{bg_{GFP}}}$$ (8)

### Calculation of growth rates at SNR of four

The SNR-light dose relationships were calculated for each possible combination of the tested fluorescent proteins with Vph1, Cdc12 or Whi5 as described (see Measurement and calculation of fluorophore brightness and signal-to-noise ratio for details). The light dose at which an SNR of four would be reached was calculated using the ”solve” function of Matlab R2015b (Mathworks, Natick, USA). Using the previously determined dose-effect relationships of photomorbidity and light dose, we were able to compute the growth rates which are expected at the respective light dose to reach an SNR of four. In the cases of mRuby2, mKO*κ*, tdKO*κ*, mKate2, mCardinal and mNeptune2, the excitation filterset for imaging (Table S6) was not identical with any filterset used to determine photomorbidity (Table S3). In these cases we based our calculations of the growth rate on the measured photomorbidity dose-effect relationship with the closest central wavelength.

The growth rates for detection of mRuby2, mKO*κ* and tdKO*κ* were based on the photomorbidity of 542/20 nm light. The growth rates for detection of mKate2, mCardinal and mNeptune2 were also determined based on the photomorbidity of 542/20 nm light, as the dose-effect relationship for red (605/15 nm) light could not be determined in *S. cerevisiae*. In these cases a conservative estimate of the growth rate is obtained, as photomorbidity for green light (542/20 nm) is more pronounced than that for red light. Extrapolating between different filtersets with comparable central wavelength is feasible, as we have shown that photomorbidity is independent of the excitation filter bandwidth for light with comparable central wavelength.

### Measurement of fluorophore photobleaching

*S. cerevisiae* cells were loaded and cultured in the microfluidic chip as described (see Measurement of fluorophore brightness and signal-to-noise ratio for details). To correct for bleaching of background fluorescence, a strain carrying no fluorescent protein tag was imaged under the same conditions. The light intensity of the fluorescence excitation light source was adjusted for all channels to be imaged with 2.91 mW cm^−2^ h^−1^.

To estimate the *in vivo* photobleaching, cells were imaged every 10 sec with an exposure time of 2 sec for 200 iterations. The resulting images were segmented (see Image analysis for detail) and the mean fluorescence intensity values ($\mu_{sig}$ and $\mu_{bg}$) were extracted (see Measurement of fluorophore brightness and signal-to-noise ratio for detail). The mean fluorescence intensity was corrected for background fluorescence ($\mu_{sig}$ - $\mu_{bg}$) and plotted against the accumulated light dose. A two-term exponential decay model (a*e^(b*x)^ + c*e^(d*x)^) was fitted using the ”fit” function of Matlab R2015b (Mathworks, Natick, USA). The light dose at which the fluorescence intensity dropped to 50% of the initial value was determined ($LD_{Bleach50}$, by solving the fitted exponential model using the ”solve” function of Matlab R2015b (Mathworks, Natick, USA).

### Data availability

File S1 contains guidelines to prevent photomorbidity in live-cell imaging in practice. File S2 contains a time-lapse movie of a growing *S. cerevisiae* colony inside the microfluidic chip. Plasmids are available from Addgene (see Table S8). Yeast strains are available upon request. The authors affirm that all data necessary for confirming the conclusions of the article are present within the article, figures, and tables. Supplemental material available at figshare: https://doi.org/10.25387/g3.13048073

## Results

### Measuring photomorbidity in time-lapse microscopy

**Figure 1:**
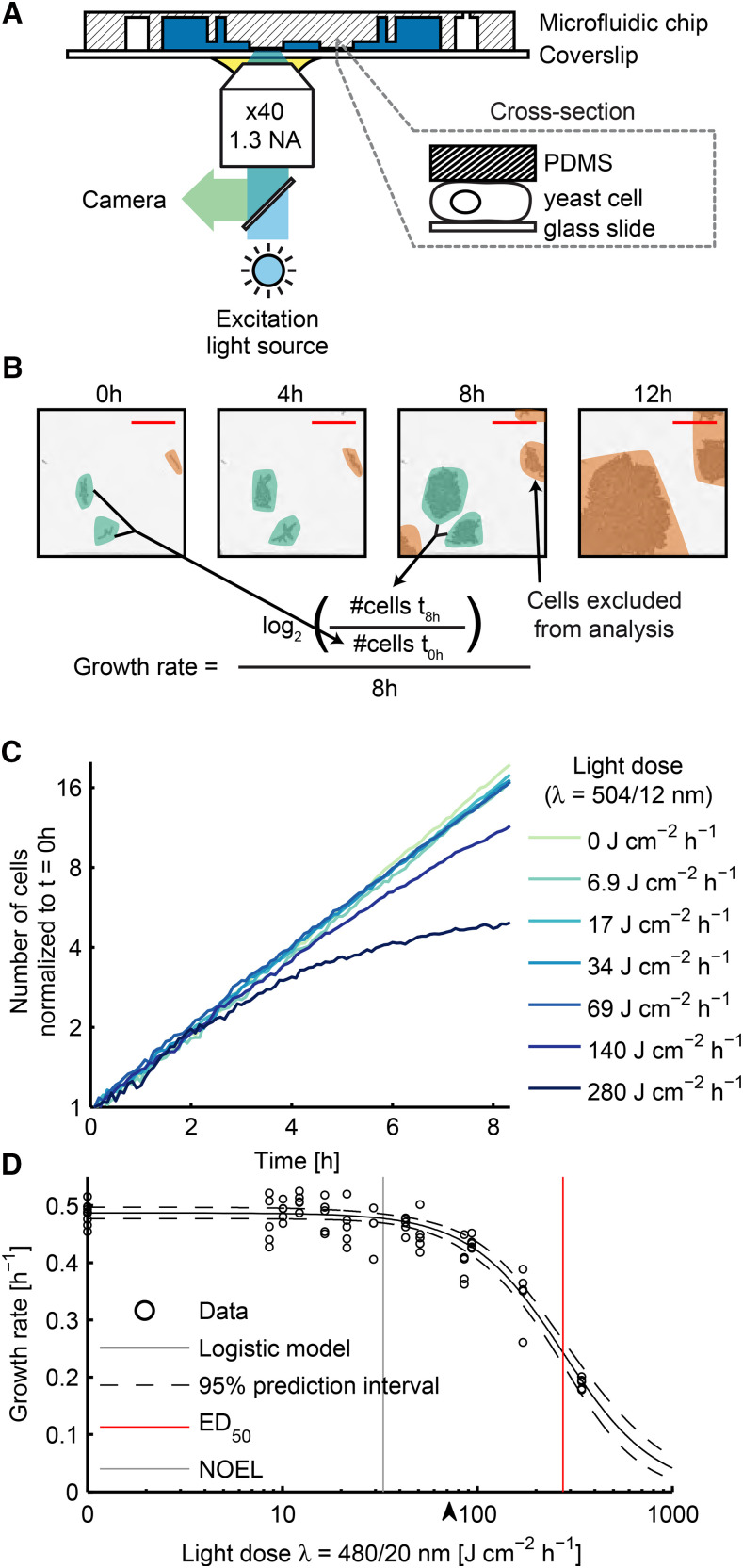
Measuring photomorbidity in a microfluidic chip using time-lapse imaging. A: Yeast cells are immobilized by clamping them between a PDMS pillar and a glass coverslip. B: Growth of *S. pombe* cells which are confined to grow in a single layer over 12 h. The GR is calculated from the number of cells at the beginning and the end of an observation period. Colonies that reach the border of the field of view (orange regions) are excluded from the analysis. C: *S. cerevisiae* growth at seven different doses of teal excitation light. For each light dose, the cell number was monitored on five independent culture pads (n = 5). Cell numbers from each condition were summed and normalized to 1 for t = 0 h. D: *S. cerevisiae* cells were exposed to different doses of cyan light. The GR obtained after 8 h was plotted against the light dose (black circles) and the sigmoidal function (equation 10) was fitted to the data (black line). Dashed lines indicate the 95% prediction interval of the fit. The ED_50_ and the NOEL are indicated by red and gray lines, respectively. The black arrow indicates the lowest light dose at which stress could be detected using the general stress transcription factor Msn2 (Logg *et al.* 2009).

The GR of a population of cells is a well established indicator for both genetic and environmental effects. Due to the magnification and limited field of view in microscopy, the largest population of cells which can be observed are small colonies and even those can suffer from nutrient limitation (Marinkovic *et al.* 2019). We implemented a population GR assay using a microfluidic chip to provide a continuous nutrient supply where *S. cerevisiae* or *S. pombe* cells grow in a single layer (adapted from (Frey *et al.* 2015)). When a cell is trapped below a PDMS pillar, all of its daughter cells are retained under the pillar and thus will form a colony (Figure 1A, File S2). After loading the microfluidic chip, we allowed the cells to adapt for at least one cell division time before we started time-lapse imaging. The average GR in each field of view can be calculated from all colonies where no cells reached the edge of the observation area (Figure 1B). The sensitivity of the assay is highest over the time period when no or only a few colonies outgrew the field of view. For glucose grown cells, we imaged in five minute intervals and realized that the maximum sensitivity is reached at ∼ 8 hr. The observed doubling time for *S. cerevisiae* is 123 ± 7 min and for *S. pombe* 128 ± 10 min (n = 5, mean ± standard deviation), which is in agreement with commonly reported doubling times in liquid culture (Sherman 2002) (Figure S1 & S2).

In a time-lapse microscopy experiment, the exposure time ($t_{E}$ in s), the light intensity (*I* in W cm^−2^), and the imaging frequency ($1/t_{Int}$ in h^−1^) can each be set individually. Their product defines the light dose per hour (LD in J cm^−2^ h^−1^, Figure S3):

$$LD=I\cdot t_{E}\cdot \frac{1}{t_{Int}}$$ (9)

To accurately determine LD, we first characterized our microscope. We measured that the sample is illuminated for an additional 187 ms compared to the set exposure time in our system (Figure S4), a hardware delay similar to previous reports (Magidson and Khodjakov 2013; Kiepas *et al.* 2020). Next, we determined the spectra and intensity of our light sources in combination with commonly used excitation filters and beamsplitters (Figure S5, Table S1 & S2). For the remainder of the text, we use the perceived color as the name for the filters: violet (*λ* = 390/18 nm), blue (438/24), cyan (480/20), teal (504/12), green (542/20) and red (600/14), and deviations of these filters are explicitly labeled. The measured intensities together with the delay corrected exposure time, and the image frequency allowed us to determine the applied light doses in each experiment.

A dose-effect curve can be used to describe the efficiency of a treatment toward a measurable observation at a specific time point. For this study, the dose-effect relationship is defined for a series of excitation light doses and the corresponding GR of the cells grown in the microfluidic device. We illuminated different culturing pads in the microfluidic device with different exposure times while keeping the light intensity and imaging interval constant (*e.g.*, seven distinct exposure times each repeated on five pads = 35 pads in one experiment). We found that the onset of growth retardation depended on the applied light dose in *S. cerevisiae*. At high doses, photomorbidity becomes visible within less than two cell division times while at lower doses, onset of photomorbidity can be delayed by several hours (Figure 1C). We therefore reasoned that the average GR from the number of cells at the beginning (t = 0 h) and end of the experiment (t = 8 h) allows for a sensitive and comparable measure of photomorbidity in cases of instantaneous and delayed growth retardation. The resulting dose-effect curve can be fitted using a sigmoidal function (Hafner *et al.* 2016):

$$GR_{\left(LD\right)}=\frac{GR_{\left(0\right)}}{1+{\left(\frac{LD}{ED_{50}}\right)}^{\tau }}$$ (10)

where ED_50_ is the effective dose at which the GR is reduced to 50% and *τ* is a measure of the capacity of cells to deal with increasing light doses (LD) once the GR is already reduced. For high values of *τ*, photomorbidity increased rapidly once a threshold dose was reached, whereas there was a slower increase in photomorbidity at small values of *τ*.

The goal of our study is to find the highest excitation light dose where no adverse effect is observed. This light dose is termed the no-observed effect level (NOEL). In our assay, small adverse effects seem to accumulate with time and are only observable at later time points (Figure 1C). We therefore fitted an exponential growth function to the first three h of each pad of the teal assay and plotted the residuals over the whole time course (Figure S6). The resulting plot showed that once photomorbidity is induced, all pads behave similarly. For practical purposes (*e.g.*, to limit the number of experiments to be conducted), the NOEL should be defined on a continuous scale. The lower confidence bound of the fitted dose-effect curve and the non-illuminated control intersects at 0.98 of the GR and this value defines the minimal experimentally observable effect. The resulting value indeed lies between the conditions where photomorbidity is observed in only a few pads and all pads and is therefore an experimentally useful definition of the NOEL. Determined in this way, the NOEL for cyan excitation light is 33 J cm^−2^ h^−1^ (Figure 1D). This is lower than the lowest light dose (72 J cm^−2^ h^−1^, black arrow in Figure 1D) reported to increase nuclear localization frequency of Msn2 measured at a comparable wavelength (*λ* = 470/40 nm) (Logg *et al.* 2009), even though it is likely that the applied light doses in (Logg *et al.* 2009) were higher than reported due to a lack of determining hardware delays.

### Morbidity is determined by the wavelength and light dose

**Figure 2:**
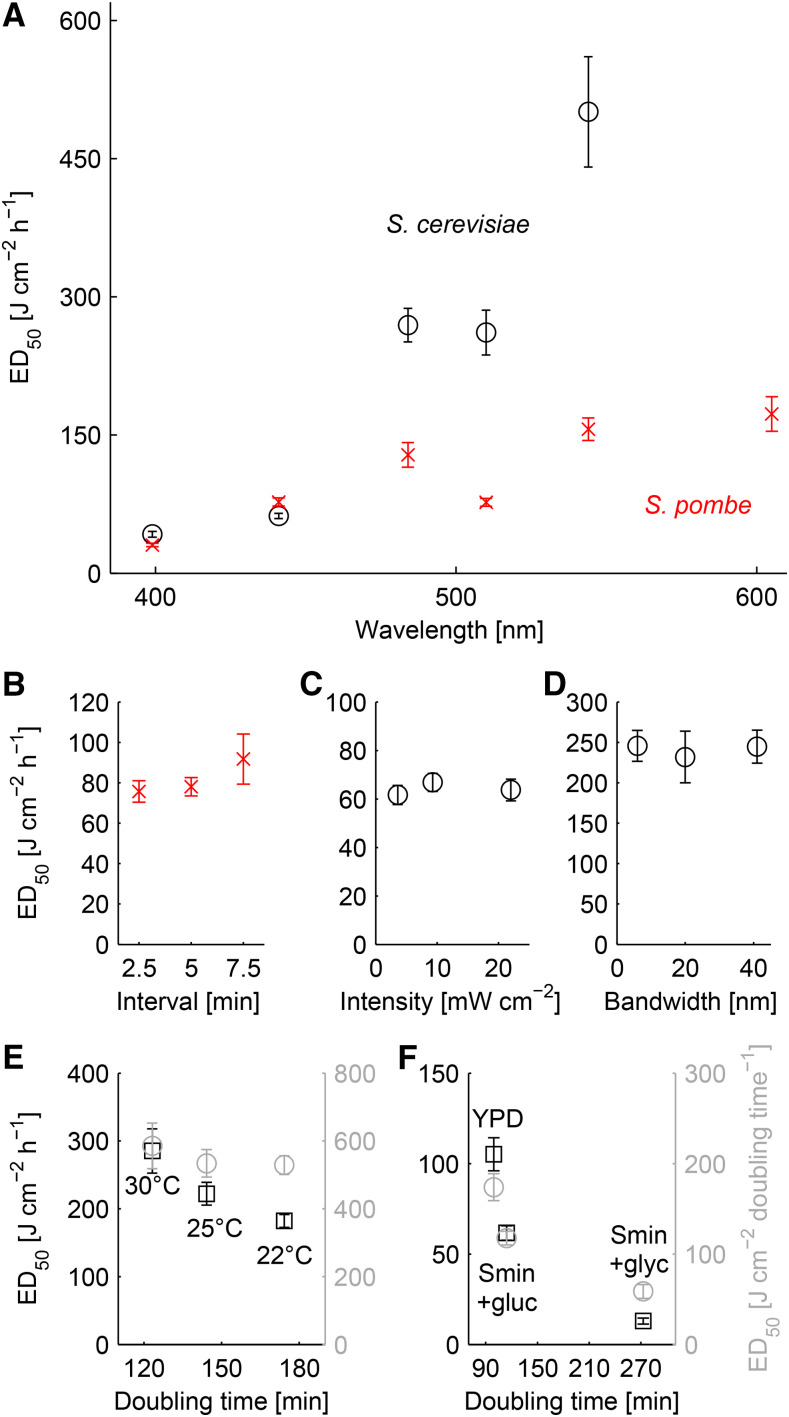
Sensitivity of *S. cerevisiae* and *S. pombe* to light using different imaging and culturing conditions. In each plot, the ED_50_-value with its 95% confidence interval are shown. Table S3 lists the optical filters and light intensities used for each measurement. A: Sensitivity of *S. cerevisiae* and *S. pombe* cells exposed to violet, blue, cyan, teal, green or red light (central wavelength according to Figure S5). B: Sensitivity of *S. pombe* to teal excitation light at imaging intervals of 2.5, 5 or 7.5 min. C: Sensitivity of *S. cerevisiae* to blue light at light intensities of 3.8, 9.5 or 22 mW cm^−2^. D: Sensitivity of *S. cerevisiae* to cyan light using excitation filters with similar central wavelength but different bandwidths (488/6 nm, 480/20 nm, 494/41 nm). The light intensity was adjusted to 3.7 mW cm^−2^ for all three excitation filters. E: Sensitivity of *S. cerevisiae* to cyan excitation light at growth temperatures of 22, 25 and 30°. The ED_50_-values are plotted as light dose per hour (gray circles) and light dose per doubling time (black squares). F: Sensitivity of *S. cerevisiae* to blue excitation light in YPD, Smin+glucose or Smin+glycerol. ED_50_-values are plotted as in (E).

We first asked at which dose excitation light of different wavelength causes photomorbidity and if they differ in the two most commonly used yeast species. We recorded the dose response as described above and plotted the ED_50_ at the different central wavelengths (Figure 2A). We used the ED_50_ to evaluate the sensitivity of cells to the applied stress as this is a characteristic measure in toxicology (IUPAC 2016). For both, *S. cerevisiae* and *S. pombe*, there is a general trend for longer wavelengths to cause less photomorbidity with the exception of teal excitation light (Figure S7 & S8). *S. pombe* appears to be more sensitive to light than *S. cerevisiae* for all wavelenghts above 480nm (Figure 2A). Photomorbidity was strongest in violet and blue light and least pronounced using green and red light. For red light applied to *S. cerevisiae*, we could not determine the ED_50_ as the low intensity of our light source led to impractically long exposure times (Table S1). It is important to note that the contribution of the brightfield illumination to the overall light dose can be neglected as it was ∼ 50-fold lower than the lowest applied fluorescence excitation light dose throughout all experiments (brightfield dose = <18 mJ cm^−2^ h^−1^, lowest fluorescence excitation light dose = 890 mJ cm^−2^ h^−1^ violet).

Different imaging settings and hardware choices may influence the dose-effect relationship. We tested whether changing the interval or the intensity of the illumination altered photomorbidity. We recorded a dose-effect curve for *S. pombe* cells excited with teal light at intervals of 2.5, 5 or 7.5 min. We found that the resulting dose-effect curves lie on top of each other (Figure S9) and the resulting ED_50_ values are the same within practical bounds (Figure 2B). We then addressed the influence of the light intensity on photomorbidity by illuminating *S. cerevisiae* cells with blue light of different intensities. Again, the obtained dose-effect curves were superimposed and ED_50_ values were similar (Figure 2C, Figure S10). We also tested the effect of the bandwidth of different excitation filters on photomorbidity. We illuminated *S. cerevisiae* cells with cyan light of similar central wavelength but different bandwidth. The obtained dose-effect curves and ED_50_ values were similar (Figure 2D, Figure S11). Previous studies indicated that using pulsed excitation light may lead to lower photomorbidity (Nishigaki *et al.* 2006; Boudreau *et al.* 2016). We determined the dose-effect relationships in *S. cerevisiae* for blue excitation light applied as a constant wave or using 5 *μ*s pulses, but did not observe a reduction in photomorbidity (Figure S12).

We also tested if the choice of the microscope objective altered photomorbidity. We recorded a dose-effect curve for *S. cerevisiae* using teal light and a 60x oil objective, which are commonly used for live-cell imaging of yeast. The apparent ED_50_ value obtained at 60x was slightly higher than the one obtained at 40x (360 *vs.* 280 J cm^−2^ h^−1^, Figure S13). Since we determined the illumination intensity without the objectives in place, we could not account for differences in light transmission between the 40x and 60x objective, which may explain a small deviation of the ED_50_ values. Nevertheless, the ED_50_ are sufficiently similar to say that photomorbidity is not influenced by the objective magnification.

Our results show that a reduction in GR is solely determined by the cumulative light dose and the utilized excitation wavelength, and is largely independent of the specific imaging settings and hardware. This is in contrast to previous reports where high light intensities were shown to induce stronger adverse effects (Dixit and Cyr 2003; Logg *et al.* 2009) than low light intensities. However, some of these contradictions may originate from hardware delays not taken into account in the aforementioned studies.

Cells with a low metabolic activity are in general more sensitive to perturbations in the environment, and it was indeed shown that photosensitivity of cells depend on their metabolic activity (Kvam and Tyrrell 1997). We set out to test whether reductions in GR due to different environmental pertubations had a similar impact on the light induced dose-effect relationships. We first slowed the growth of *S. cerevisiae* by lowering the temperature from 30° to 25° or 22° and illuminated them with cyan excitation light. The ED_50_ values were 290, 220 and 180 J cm^−2^ h^−1^, respectively (gray circles, Figure 2E, Figure S14) which indicates a higher sensitivity of cells at lower temperatures as expected. However, when we normalized the applied light doses to the doubling time at the respective growth temperature, the ED_50_-values became comparable at 590 ± 67, 530 ± 40 and 530 ± 27 J ${\text{cm}}^{-2}$ doubling ${\text{time}}^{-1}$ (black squares, Figure 2E, Figure S14). This indicates that cells are able to cope with a defined amount of light induced damage during a cell cycle, in case the GR is perturbed by a change in temperature but the chemical composition of the environment is comparable.

Last but not least we asked what happens when the GR is altered through a change in the chemical composition of the media. We compared the photosensitivity of cells grown in rich or minimal media supplemented with glucose, and cell grown in minimal media with the non-fermentable carbon source glycerol. Glycerol grown cells were twice as sensitive to blue light compared to glucose grown cells, even after normalization of the light dose to the doubling time (59 *vs.* 120 J cm^−2^ doubling time^−1^, black squares, Figure 2F, Figure S15). Additionally, cells grown on glucose were more sensitive to light in minimal media compared to rich media (120 *vs.* 170 J cm^−2^ doubling time^−1^, black squares, Figure 2F, Figure S15). These differences can be rationalized by changes in the expression of endogenous photosensitizers, like porphyrins, whose presence correlates with photosensitivity in *S. cerevisiae* (Strakhovskaya *et al.* 1999) and which are upregulated in growth on non-fermentable carbon sources (Roberts and Hudson 2006). Depending on the absorption spectra of such photosensitizers the relative susceptibility at different wavelengths can change, as is the case for susceptibility to teal excitation light of cells grown in glycerol compared to glucose, which was even higher (170 *vs.* 550 J cm^−2^ doubling time^−1^, Figure S16).

### Detectability of fluorescent proteins at NOEL light doses

The goal of live cell imaging is to obtain a detectable signal without damaging the cell. The obtained signal depends on the brightness of the fluorescent reporter and the amount of cellular autofluorescence (Pang *et al.* 2012). We therefore judged the usability of different fluorescent proteins using the SNR (Waters 2009):

$$SNR=\frac{\mu_{sig}-\mu_{bg}}{\sqrt{\mu_{sig}}}$$ (11)

where $\mu_{sig}$ is the fluorescent signal, $\mu_{bg}$ the background fluorescence and $\sqrt{\mu_{sig}}$ an estimate of the statistical fluctuations in the measurement. The fluorescent signal:

$$\mu_{sig}=\left(B+P\right)\cdot t_{E}\cdot I\cdot Q_{E}+N_{R}^{2}$$ (12)

is composed of the photons originating from the fluorescent probe (*P*) and the background fluorescence (*B*, cellular autofluorescence and fluorescence of media) as well as contributions from the noise of the camera ($N_{R}^{2}$) (Pang *et al.* 2012). The magnitude of *P* and *B* depend on the quantity and brightness, and by how well the wavelength and bandwidth of the optical filters match the excitation and emission spectra of the fluorescent molecules. Additionally, $\mu_{sig}$ depends on the quantum efficiency of the detector ($Q_{E}$) in the associated spectral region and the exposure time ($t_{E}$) and light intensity (*I*).

**Figure 3:**
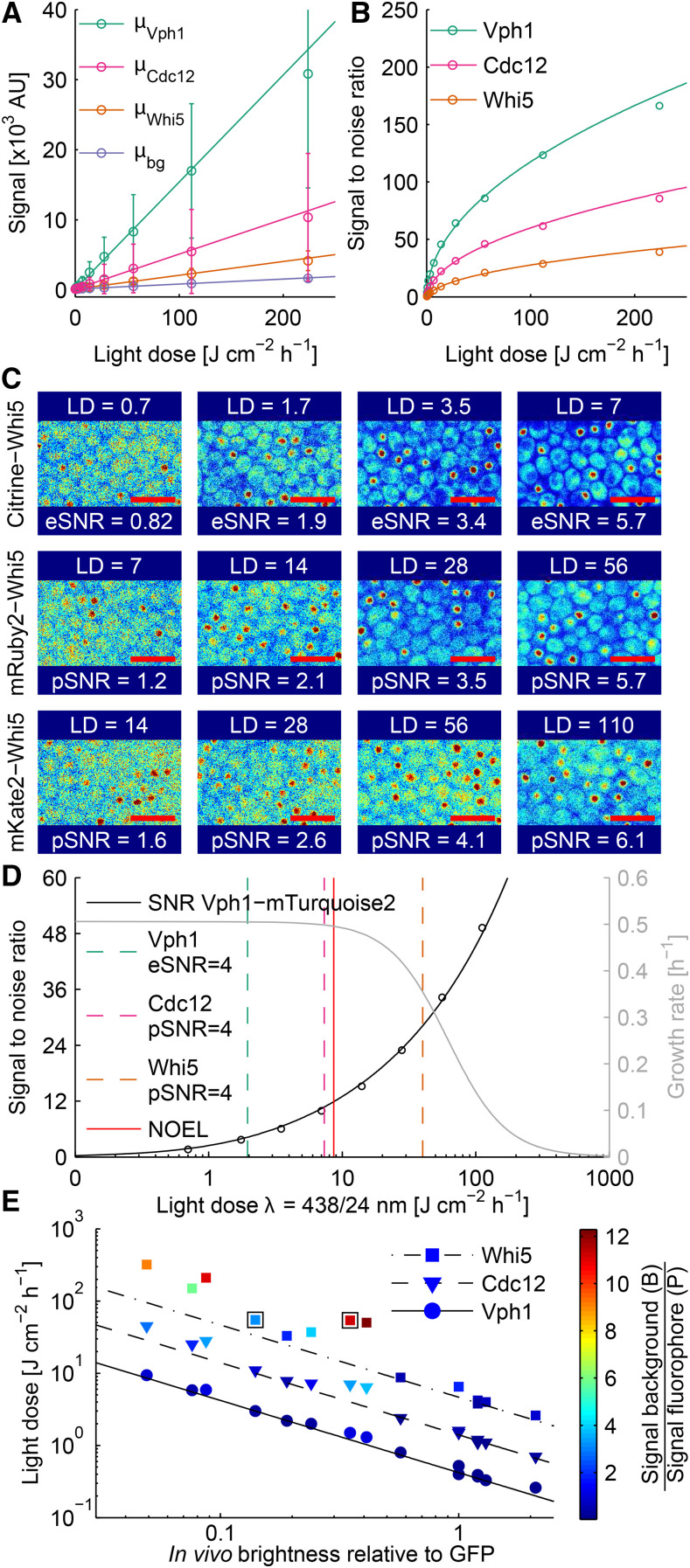
Detectability of fluorescent proteins at their respective NOEL in *S. cerevisiae*. A: Signal intensity *vs.* excitation light dose for indicated strains tagged with Citrine and a wildtype strain. The mean ± SD of the cellular fluorescence is plotted. Points denote measurements and lines indicate linear fit. B: SNR *vs.* light dose plot. Points denote measured SNR values (mSNR) calculated using the single measurement points from A according to equation 11. Lines are the estimated SNR values (eSNR) calculated from the respective linear fit. C: Contrast adjusted false color fluorescent images of Whi5 strains tagged with different proteins at increasing light doses (LD in J cm^−2^ h^−1^). For Citrine the eSNR is shown while for mRuby2 and mKate2 the pSNR (predicted SNR based on measurements of Vph1-tagged strains, see text for details) is shown. Images are contrast-adjusted such that 1% of the highest and lowest pixel values are saturated. Red scale bars indicate 10 *μ*m. D: SNR and photomorbidity *vs.* LD per hour for a Vph1-mTurquoise2 strain imaged with blue light at an interval of five min. Dashed green line indicates an eSNR of four for Vph1, while dashed red and orange lines indicate pSNR of four for Cdc12 and Whi5, respectively. NOEL is indicated by a red line. E: LD required for an SNR of four *vs.* *in vivo* brightness for all tested fluorescent fusion proteins. The data points are colored according to the ratio of the background signal (untagged strain) divided by the fluorescent signal (tagged strain). The two boxes indicate the data points for Whi5-mKate2 and Whi5-mAmetrine. Data are listed in Table 1.

The fluorescent signal is a combination of all its different components which should increase linearly with increasing excitation light dose. We verified this linear relationship by growing cells in our setup and quantifying the fluorescent signal at different excitation light doses form yeast strains expressing Citrine as a C-terminal fusion to proteins expressed at different levels (Figure 3A). We calculated the *in vivo* brightness ($\mu_{sig}$ - $\mu_{bg}$) to be 143 (Vph1 - subunit of the vacuolar ATPase V0 domain), 43 (Cdc12 - a septin component) and 13 (Whi5 - a transcriptional repressor) AU J^−1^ cm^2^ h. The ratio of the brightness of those three strains should be independent of the used fluorophore, but depend only the expression level of the tagged proteins. We confirmed this by measuring the brightness of strains where the same three proteins where tagged with either mRuby2 or mKate2. The ratio is indeed constant as long as $\mu_{sig}$ is discernible from $\mu_{bg}$ (Figure S17, S18 & S19). Therefore, the signal intensity for different target proteins can be estimated in cases where their relative abundances have been determined.

The SNR describes the quality of an image, and can be calculated if $\mu_{sig}$ and $\mu_{bg}$ are known (Equation 11). We reasoned that the SNR should be predictable, as it is limited by ”camera noise” at low light doses as $\mu_{sig}$ is dominated by the readout noise of the camera ($\left(P+B\right)\cdot t_{E}\cdot I\cdot Q_{E}<<N_{R}^{2}$). Once the signal is comparable to the noise, the SNR becomes limited by stochastic fluctuations in the fluorescent signal $\sqrt{\mu_{sig}}$ (”photon or shot noise”) (Lambert and Waters 2014). To verify that the SNR is indeed calculable for any light dose, we estimated the SNR (eSNR) using the slopes of $\mu_{sig}$ and $\mu_{bg}$ and compared it to the measured SNR (mSNR) values we obtained directly from images. The eSNR (lines) calculated from the slopes are in good agreement with the mSNRs (circles, Figure 3B), and higher light doses as well as higher protein expression levels yield higher SNRs as expected. However, it is not feasible to measure $\mu_{sig}$ for any combination of target protein and fluorophore. We therefore tested if we can predict the SNR (pSNR) for strains carrying Whi5-mRuby2 and Whi5-mKate2 from measurements of Vph1-mRuby2 and Vph1-mKate2 strains. We estimated $\mu_{whi5}$ from $\mu_{vph1}$ using the relative abundance ratio obtained from Whi5-Citrine and Vph1-Citrine fusions (from Figure 3A). Based on this we calculated the pSNR for Whi5-mRuby2 and Whi5-mKate2 at different light doses. We found a reasonable agreement between the eSNR and pSNR values of images with optically comparable quality (Figure 3C - first row shows eSNR, bottom two rows show pSNR). These results show that the light dose dependent image quality for a given fusion protein can be estimated, if $\mu_{sig}$ and $\mu_{bg}$ of a reference strain and the relative abundance of the fusion proteins are known.

This enabled us to identify those fluorescent proteins that are able to provide sufficient signal without damaging the cell, by predicting the light dose required to reach a certain SNR for each combination of fluorophore and target protein. We decided to use an SNR of four to compare the performance of commonly used fluorescent proteins, since in our experience this is the lowest SNR at which we can obtain robust quantitative data of the cellular fluorescence signal (see Figure 3C for image quality). We first measured the background corrected *in vivo* brightness of different fluorescent proteins for a set of Vph1-tagged strains ($\mu_{vph1}$). We then calculated the light doses required to reach an SNR of four and extracted the expected GRs from the photomorbidity curves (Figure 3D, Figure S20, Table 1). As we could not determine a dose-effect curve for red excitation light, we utilized the data for green excitation light which leads to an overestimation of photomorbidity. All tested Vph1 fusions could be detected at light doses below the NOEL in *S. cerevisiae*. In contrast, Whi5 could only be detected using fusion to GFP, mNeonGreen, Citrine, mRuby2, mKO*κ* and mKate2 fluorescent proteins without inducing photomorbidity. Additionally, mCardinal and mNeptune2 allowed imaging of Cdc12 at NOEL light doses.

**Table 1:** Properties of Vph1-fusion proteins

Fluorescent	Excitation	Emission	Relative*^a^*	*μ_bg_**^b^*	LD*_vph1_^C^*	LD*_cdc12_*	LD*_whi5_*	GR*_vph1_**^d^*	GR*_cdc12_*	GR*_whi5_*	LD*_Bleach50_**^e^*
protein	filter	filter	brightness	at SNR = 4				at SNR = 4			
tSapphire	390/18	525/50	41	54	1.3	6.4	50	100	99	40	3010
mAmetrine	438/24	525/50	35	43	1.5	7.0	54	100	99	57	6320
mTFP1	438/24	480/17	8.7	10	5.9	28	210	99	83	0.79	866
mTurquoise2	438/24	480/17	24	10	2.0	7.2	37	100	99	73	21200
sfGFP	488/6	525/50	100	20	0.4	1.6	6.5	100	100	100	5940
eGFP	488/6	525/50	100	20	0.52	1.5	6.5	100	100	100	15200
mNeongreen	488/6	525/50	210	20	0.26	0.7	2.6	100	100	100	6520
mNeongreen	504/12	542/22	120	8.8	0.37	1.2	4.2	100	100	100	3730
CitrineA206K	504/12	542/22	130	8.8	0.33	1.1	4.0	100	100	100	633
mRuby2	561/4	600/32	19	3.8	2.2	7.8	33	100	100	100	5320
tdKOkappa	546/10	577/25	120	3.1	0.39	1.1	3.8	100	100	100	4200
mKO*k*	546/10	577/25	57	3.1	0.80	2.4	8.7	100	100	100	7430
mKate2	600/14	655/40	14	4.8	3.0	11	55	100	100	100	2930
mCardinal	600/14	655/40	7.6	4.8	5.8	25	150	100	100	100	7320
mNeptune2	600/14	655/40	4.9	4.8	9.4	45	320	100	100	>89	9100

a given in [%]

b given in [AU h J^-1^ cm^2^]

c given in [J cm^-2^ h^-1^], based on imaging interval of 5 min.

d given in [%], based on imaging interval of 5 min.

e given in [J cm^-2^]

The obtainable SNR also depends on the magnification and numerical aperture (NA) of the microscope objective (Rines *et al.* 2011; Frigault *et al.* 2009). Many yeast researchers work with 60x objectives as opposed to the 40x objective used in our study. In theory, the SNR should be proportional to NA^4^ and inversely proportional to the square of the objective magnification. We tested if this relationship holds true for our SNR measurements, by using a 60x objective with comparable NA as our standard 40x objective. Use of such a 60x objective should decrease the SNR by a factor of 2.25. Indeed, the SNR measured with the 60x objective decreased approximately two-fold as compared to the 40x objective. This means that significantly higher light doses are required to reach a detectable signal with a 60x objective (Figure S21).

We noticed that Whi5-mKate2 and Whi5-mAmetrine yield similar SNRs at almost identical light doses (55 and 54 J cm^−2^ h^−1^), although mKate2 is only 40% as bright as mAmetrine. The main difference between the two imaging channels is the amount of cellular background fluorescence ($\mu_{bg}$, 43 and 4.8 AU J^−1^ cm^2^ h, Table 1).To better understand the interplay between fluorescent signal (*P*) and background signal (*B*), we plotted the light doses necessary to reach SNR = 4 against the *in vivo* brightness normalized to GFP (Figure 3E). The two measures were inversely correlated for an abundant protein like Vph1. In contrast, the inverse correlation is lost when *B* is more than three times larger than *P* (see colorbar in Figure 3E).

Knowing that the background fluorescence plays a major role in determining the SNR, we tried to find the best prediction for the amount of autofluorescence in a given imaging channel (Figure S22A). We found that the background fluorescence corrected for the bandwidth of the emission filter strongly correlated with the excitation wavelength (R2 = 0.92, Figure S22B). Therefore, the width of the emission filter can be used to optimize the ratio of *B* to *P*. Surprisingly, we found that the bandwidth corrected *B* is similar for channels excited at the same wavelength (compare Ex390/18 Em460/50 *vs.* Em525/50 and Ex438/24 Em480/17 *vs.* Em525/50, Figure S22C). However, as the background fluorescence of cells is influenced by the culturing conditions (Heppert *et al.* 2016) and the imaging setup, its magnitude needs to be determined experimentally.

### Predicting no-observed effect level imaging conditions in multi-color imaging

Excitation light can be seen as stress that has a quantifiable effect on the cell. However, the interaction of several such effects in multi-color imaging is difficult to predict. Currently, there are no studies on how two or more separate excitation wavelengths interact with cellular physiology. To address whether photomorbid effects act independent of each other, we exposed *S. cerevisiae* to combinations of two different excitation wavelengths. As a score for the interaction, we used the coefficient of drug interaction (CDI) which is a common pharmacological measure for the interaction of two effects. The CDI is defined as:

$$CDI=\frac{{\text{effect}}_{combined}}{{\text{effect}}_{1}\cdot {\text{effect}}_{2}}=\frac{{\text{GR light}}_{AB}}{{\text{GR light}}_{A}\cdot {\text{GR light}}_{B}}$$ (13)

**Figure 4:**
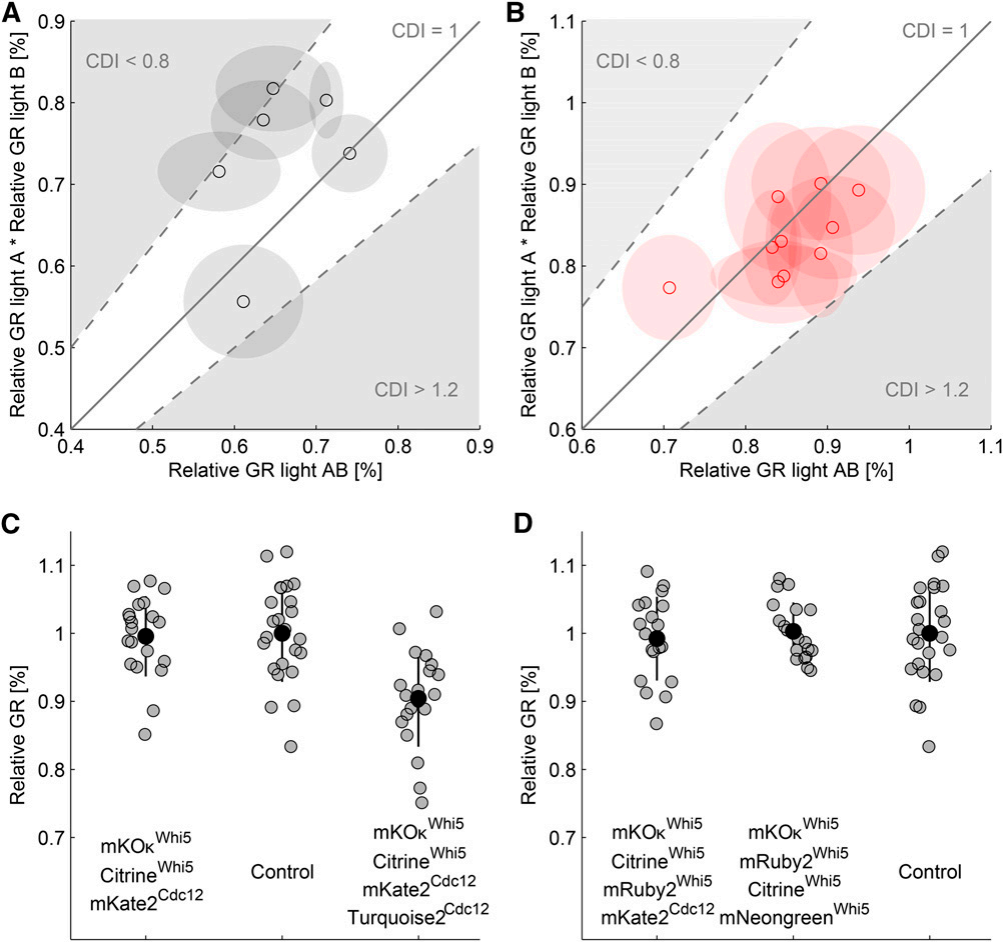
Photomorbidity is additive. A: Normalized *S. cerevisiae* GRs obtained during dual-color illumination with pairwise combinations of morbid light doses (GR ${\text{light}}_{\mathrm{AB}}$) were plotted against the product of the respective single color treatments (GR light_*A*_ ⋅ GR light_*B*_). Each point is the mean of five replicates and shaded circles denote standard deviations. For clarity, the line shows an additive model (CDI = 1) and the gray area where the CDI is either <0.8 or >1.2 (complete dataset in Figure S23). B: Same as in (A) but with *S. pombe* (complete dataset in Figure S24). C: NOEL imaging under the assumption of additive toxicity using four distinct excitation wavelength. The mean normalized GR (black dots), 20 individual measurements (gray dots) and standard deviation (black lines) are plotted against the combination of the utilized fluorescence channels. The indication shows which target protein can be detected at an SNR of four. D: NOEL imaging of four low expressed proteins when allowing for overlapping emission spectra. Axes as in (C).

Effects exhibiting additive behavior are considered independent (CDIs ≈ 1), whereas synergistic or antagonistic behaviors yield CDIs of <0.8 or >1.2, respectively (Bijnsdorp *et al.* 2011). For example, we observed a GR reduction to 74% when combining blue and teal light doses that, as single color doses, yielded GR reductions to 85% and 87% of the control, respectively. In this particular case the CDI is 1. Also for other pairwise combinations, photomorbidity seems to be additive since all of them yielded CDIs between 0.79 and 1.1 (Figure 4A, Figure S23). We confirmed this observation by using pairwise combinations of weakly morbid light doses in the more photosensitive yeast *S. pombe*. This resulted in CDIs between 0.91 and 1.1 (Figure 4B, Figure S24). These results show that doses of excitation light relevant for non-toxic imaging act independently.

Also in multi-color imaging, we defined the NOEL as the smallest detectable reduction in growth rate (98% of control GR). The additive effects of all used wavelengths should not reduce the GR any further. Additivity implies that the multi-color NOEL for each individual wavelength can be calculated as the *n*th root of 0.98, where *n* denotes the number of distinct excitation wavelengths. We tested for the absence of photomorbid effects in three-color imaging (LDs for each excitation wavelength allow GRs >$\sqrt[{3}]{0.98}$ = 0.993) by applying combinations of excitation light doses aimed to detect a set of low abundant proteins in *S. cerevisiae*. First, we used a combination of three fluorophores with negligible emission cross-talk (Citrine, mKO*κ* and mKate2) and simulated detection of two proteins expressed at the Whi5-level and one protein at the Cdc12-level each at an SNR of four. This combination resulted in GRs indistinguishable from the control (Figure 4C). Next, we tested if we were accurately predicting the maximum available light dose for each single channel by adding a fourth light dose (mTurqouise with a relative GR of 0.99) and thereby slightly exceeding the predicted four-color NOEL. As expected, the resulting GR was reduced below the NOEL to 91% of the control.

A possible way to extend multi-color NOEL imaging would be to combine fluorescent proteins that are excited in spectral regions were cells are able to tolerate high doses of light. As a proof of concept, we chose to simulate the imaging of four low abundant proteins using long excitation wavelengths only. In this case, we had to allow for crosstalk of the respective emission spectra as well as using excitation wavelength bands which lie within the same photomorbidity window. The simulated combination (mKO*κ*, mRuby2, mKate2 and Citrine) uses three distinct excitation bands (mKO*κ* and mRuby2 are excited within the green window). This combination would allow for NOEL imaging of three proteins at the Whi5-level and one at the Cdc12-level (Figure 4D). Similarly, four low abundant proteins (Whi5-level) could be imaged using pairs of bright proteins excited with the same excitation wavelength band and substantial spectral overlap (Figure 4D). Our results indicate that imaging using unconventional combinations of well performing fluorescent proteins has the potential to detect more low abundant targets without inducing photomorbidity. However, such experiments would require the use of spectral unmixing algorithms, due to the inherent emission crosstalk of the available fluorophores (Zimmermann 2005).

## Discussion

To develop guidelines to avoid the confounding effects of visible light in multi-color long term imaging using fluorescent proteins, we first defined and characterized a reliable, sensitive and label-free measure based on slowed cell growth (photomorbidity). We propose to use the term ”photomorbidity”, defined as the adverse effect of light on cellular well being as measured by a decrease in growth rate compared to an unstressed control, to describe subtle non-toxic effects of excitation light on dividing cells. This is consistent with the definition of ”morbidity” by IUPAC: ”departure, subjective or objective, from a state of physiological or psychological well-being” (IUPAC 2016) and distinct from the more severe term ”phototoxicity”, which is used to describe cell death. We found that photomorbidity is specific for each species, wavelength, and metabolic condition and its onset can be delayed by several hours. Our results show that the sensitivity to growth inhibition by visible light depends on the light dose, wavelength, and metabolic state determined by the growth medium. For a given set of conditions, our results point to a tolerable light dose per cell cycle which is independent of the light intensity, imaging interval and bandwidth of the used excitation light.

Photomorbid effects of several wavelengths are additive, and hence NOEL light doses for multi-color imaging are different than those for single-color imaging. The multi-color NOEL doses for each excitation wavelength can be calculated based on the respective photomorbidity curve. Together with the predicted light dose to reach a defined SNR, it is possible to determine a combination of fluorescent proteins that will allow unstressed imaging. Use of fluorescent reporters excited with short excitation wavelengths (<480 nm) should be limited to strongly expressed proteins or avoided completely whenever possible. Alternatively, wavelengths which cells are able to tolerate at large doses can be used for the detection of fluorescent proteins with overlapping excitation spectra. We expect that these rules and our approach to establish unstressed imaging conditions can be applied to any dividing specimen and other imaging technologies. A detailed discussion of the practical aspects can be found in File S1.

The choice of fluorescent proteins governs if a target protein can be detected with the required SNR at doses below the NOEL. This choice is based on the brightness of the fluorescent protein, the autofluorescence in the respective imaging channel and the photomorbidity exerted by the excitation light. Of course, the suitability of a fluorescent protein additionally depends on properties like oligomerization tendency, pH stability or maturation time (Cranfill *et al.* 2016). However, we demonstrate for the tested fluorescent proteins that photostability can be neglected in long-term imaging of yeast as the light doses which lead to significant bleaching are above the NOEL (Table 1). The detectability of a fluorescent reporter can be optimized by choosing emission filters such that the contribution of the signal is maximized compared to the contribution of the background. Narrow excitation filters at the excitation maximum of the fluorophore can be used to optimize the excitation efficiency as photomorbidity is independent of the excitation light bandwidth. Bright and well behaved fluorescent proteins with different stokes shifts emitting in the orange and red part of the spectrum would further facilitate unstressed multi-color imaging by replacing fluorescent reporters excited at strongly photomorbid short wavelengths.

A large part of our results quantify previously held beliefs in the field of fluorescence time-lapse imaging and allow for simple improvements of established assays. Probably the easiest and most cost effective way is the replacement of standard excitation and emission filters with customized filters for a specific fluorophore and application. As a rule of thumb, one should use narrow (<20 nm) bandpass filters close to the excitation and emission maximum. Additionally, the results point out under which conditions extra care has to be taken to ensure NOEL imaging. This is especially cumbersome for assays requiring UV-A excitation light, high magnification or poor growth conditions. Some of our results contradict previous studies, especially the absence of a correlation between photomorbidity and the intensity of the excitation light (Dixit and Cyr 2003; Logg *et al.* 2009) as well as a lack of effects on photomorbidity and background fluorescence from pulsed illumination regimes (Nishigaki *et al.* 2006; Boudreau *et al.* 2016). Some of these discrepancies are most likely explained by unaccounted for hardware delays, as has been shown recently (Kiepas *et al.* 2020). However, our inability to confirm the benefits of pulsed illumination will require further investigation. Last, our reasoning was developed by studying dividing yeast cells but it can be easily translated to any other dividing cell type.
